# New predictive models for falls among inpatients using public ADL scale in Japan: A retrospective observational study of 7,858 patients in acute care setting

**DOI:** 10.1371/journal.pone.0236130

**Published:** 2020-07-16

**Authors:** Masaki Tago, Naoko E. Katsuki, Yoshimasa Oda, Eiji Nakatani, Takashi Sugioka, Shu-ichi Yamashita

**Affiliations:** 1 Department of General Medicine, Saga University Hospital, Saga, Japan; 2 Department of General Medicine, Yuai-Kai Foundation and Oda Hospital, Saga, Japan; 3 Division of Statistical Analysis, Research Support Center, Shizuoka General Hospital, Shizuoka, Japan; 4 Translational Research Center for Medical Innovation, Foundation for Biomedical Research and Innovation at Kobe, Hyogo, Japan; 5 Community Medical Support Institute, Faculty of Medicine, Saga University, Saga, Japan; Universita degli Studi di Napoli Federico II, ITALY

## Abstract

**Aim:**

Most predictive models for falls developed previously were awkward to use because of their complexity. We developed and validated a new easier-to-use predictive model for falls of adult inpatients using easily accessible information including the public ADL scale in Japan.

**Methods:**

We retrospectively analyzed data from Japanese adult inpatients in an acute care hospital from 2012 to 2015. Two-thirds of cases were randomly extracted to the test set and one-third to the validation set. Data including age, sex, activity of daily living (ADL), public scales in Japan of ADL “bedriddenness rank,” and cognitive function in daily living, hypnotic medications, previous falls, and emergency admission were derived from hospital records. Falls during hospitalization were identified from incident reports. Two predictive models were created by multivariate analysis, each of which was assessed by area under the curve (AUC) from the validation set.

**Results:**

A total of 7,858 adult participants were available. The AUC of model 1, using 13 factors—age, sex (male), emergency admission, use of ambulance, referral letter, admission to Neurosurgery, admission to Internal Medicine, use of hypnotic medication, permanent damage by stroke, history of falls, visual impairment, independence of eating, and bedriddenness rank—with low mutual collinearity and showing significant relationship by multivariate logistic regression analysis, was 0.789 in the validation set. The AUC of parsimonious model 2, using age and seven factors—sex (male), emergency admission, admission to Neurosurgery, use of hypnotic medication, history of falls, independence of eating, and bedriddenness rank—showing statistical significance by multivariate analysis in model 1, was 0.787 in the validation set.

**Conclusions:**

We proposed new predictive models for inpatients’ fall using the public ADL scales in Japan, which had a higher degree of usability because of their use of simpler and fewer (8 or 13) predictors, especially parsimonious model 2.

## Introduction

Falls can be devastating events leading to severe injuries [1], restriction of activities [2], or reduced activities of daily living (ADLs) [3]. The economic burden of medical costs related to severe injuries caused by falls is high [4], which is partly the cause of ballooning national medical expenses in the rapidly aging society of Japan [5]. Usually inpatients have a higher frequency of falls because the population includes a higher proportion of older people >65 years old who are prone to fall [6] or those with some disability such as limb weakness [7]. Among inpatients, those admitted to acute care hospitals particularly have an extraordinarily high frequency of falls (3.15–4.18 per 1,000 patient-days) [8]. Additionally, because falls by inpatients can potentially lead to medical lawsuits [9], it is imperative to take measures to prevent falls in acute care settings.

Previous community-based prospective cohort studies have identified a variety of risk factors for falls, such as history of a fall [7, 10], lower extremity weakness [7, 10], older age [6, 11, 12], female sex [11], cognitive impairment [6, 10, 11], balance problems [10, 11], use of psychotropic drugs [11], arthritis [10, 11], history of stroke [10, 12], orthostatic hypotension [10, 11], dizziness [11], syncope [13], and nocturia [14, 15]. In addition to such risk factors, several predictive models for falls have been developed, including the Morse Fall Scale [16], St Thomas Risk Assessment Tool in Falling Elderly Inpatients [17], Tinetti mobility test [18], and Hendrich II Fall Risk Model (HFRM) [19]. Although the HFRM was reported to be the most appropriate predictive model for inpatients in acute care hospitals [20], it requires examinations and techniques seldom used in routine clinical settings as well as time-consuming assessment items, which pose a serious impediment to its use. Therefore, we developed predictive models, less complicated to use and with acceptable and satisfactory accuracy, using more readily available information routinely obtained on admission to commonplace Japanese hospitals, which were subsequently validated.

Some studies showed that poor ADL is a risk factor for falls. Bedriddenness rank is a public measure of ADL provided by Japan's Ministry of Health, Labour and Welfare (MHLW), widely used in Japan's long-term care insurance system, which can be easily assessed by the degree of confinement of a patient in daily life, such as at home, in a chair, or in a bed, whereas no fall prediction model using this rank has ever been presented.

We herein report two predictive models that, by the use of public ADL classifications evaluated on admission of falls, are far easier to use than others previously developed.

## Materials and methods

### Study design and population

This was a retrospective cohort study using medical charts. All inpatients of age ≥20 years who were admitted to an acute care hospital (Yuai-kai Foundation and Oda Hospital) (S1 Appendix) between April 2012 and January 2015 were included.

### Data

The data in this study were extracted from the hospital’s health records as follows. The variables on admission were age, sex (male and female), department of admission (Internal Medicine, Neurosurgery, or others), emergency admission (presence or absence), ambulance transfer (presence or absence), ADLs (ability to eat, go to the toilet, bathe, take prescribed medicines, and move independently), MHLW classifications for abilities of daily living, use of hypnotic medications (both benzodiazepine and non-benzodiazepine types; presence or absence), dependence on a wheelchair (presence or absence), admission with a referral letter from a primary physician (presence or absence), permanent residual damage from previous strokes, visual impairment, parkinsonism (presence or absence), and a history of falls (presence or absence).

The possible candidates of risk factors for falls and fall injuries were selected from previously reported community-based prospective cohort studies (e.g., old age, female gender, cognitive impairment, use of a hypotonic medications, history of stroke, parkinsonism, visual impairment, limitations in ADLs, and cognitive impairment [7, 10, 11]). Additionally, information on the employment of preventive measures for falls (fall preventive movement sensor, low bed, and impact-absorbing mat), undergoing a surgical operation, rehabilitation during hospitalization, and outcome at discharge (deceased or alive) was also extracted.

Variables relating to the presence of difficulties in ADLs were checked on admission by medical personnel, mainly nurses. Some patients’ ADLs had not been assessed on admission because of their normal-appearing features and behaviors, being categorized as “normal ADLs.” Although the actual individual degree of “visual impairment” was not clearly defined in this retrospective study, attending nurses evaluated whether visual disorder impaired a patient’s daily life. Although “fall” had not been defined clearly, falls were identified from the fall-specific report forms in the incident reports, which were mandatorily recorded and submitted by attending medical personnel when falls occurred during a hospital stay. Falls in the incident reports included any unplanned descent of a patient to the floor with or without suffering injuries [21], from any height and in any position; i.e., on the floor, from stairs, chair, or bed, when standing, walking, or sitting, or even in recumbent position.

### MHLW bedriddenness rank and cognitive function scores: ADL classifications for abilities of daily living

**Fig 1:**
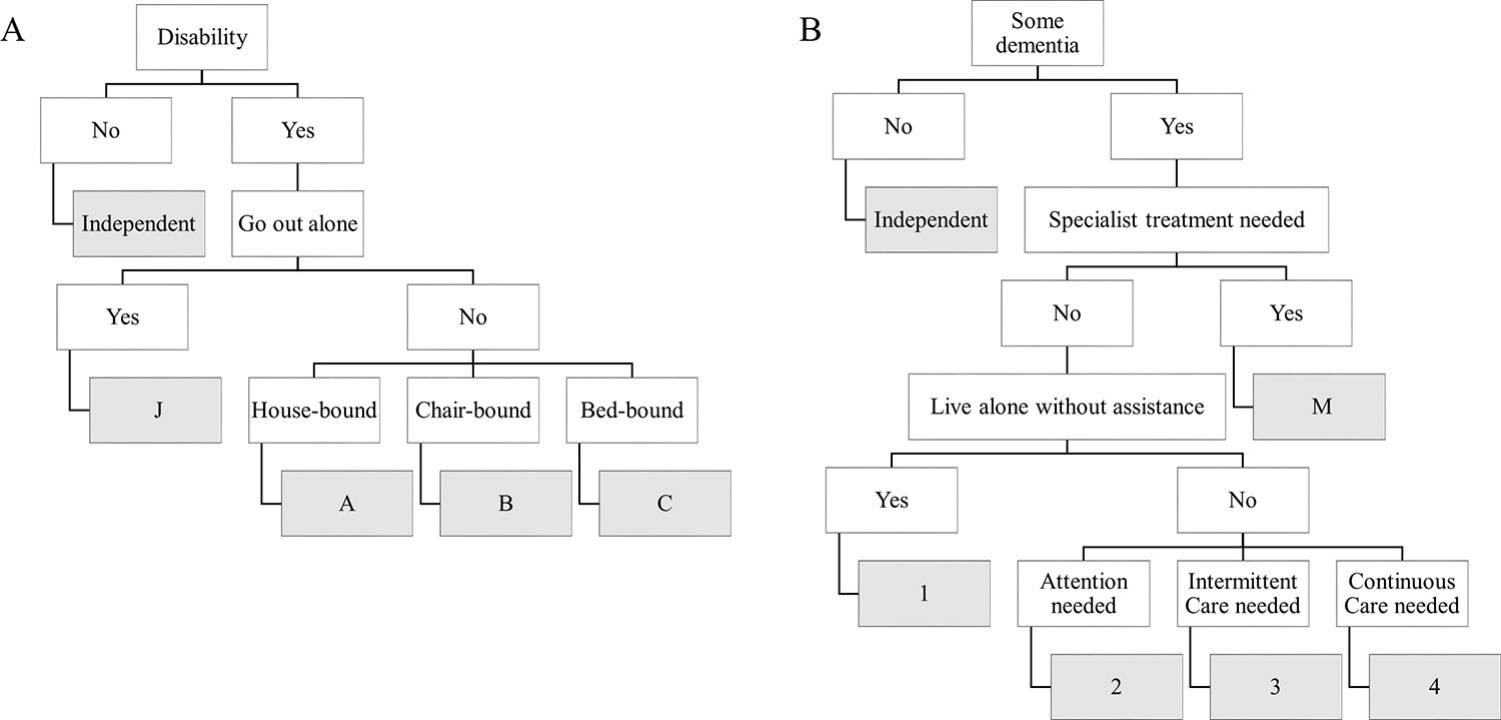
The Japan MHLW bedriddenness rank and cognitive function score in daily living. Bedriddenness rank is assessed by the degree of confinement of daily life of a patient; at home, in a chair or a bed (A). Cognitive function score is determined by whether the patient has difficulties in communication or problematic behaviors requiring specialist treatment, and how often they have those features (B). MHLW, Ministry of Health, Labour and Welfare.

MHLW bedriddenness rank and cognitive function scores are public assessment tools used in the Japanese long-term care insurance system, and are also used to help patients prepare their living conditions upon discharge from medical facilities in Japan [22, 23]. Bedriddenness rank was easily assessed by the degree of confinement of daily life of a patient; at home, in a chair, or in a bed (Fig 1A). Cognitive function scores were determined by whether difficulties were encountered in communication or problematic behaviors, and how often they featured (Fig 1B). MHLW bedriddenness rank and cognitive function scores were routinely assessed by medical personnel, mainly nurses, on admission while providing them with care, or were gathered as reported by their family members.

### Statistical analysis

Continuous and categorical variables were presented as median (interquartile) and number (percentage). Included cases were randomly divided into the test set and validation set at a ratio of 2:1. Univariate and multivariate logistic regression analyses for fall events were performed, with odds ratio, 95% confidence interval (CI), and p value (based on the Wald test) being calculated. The factors that were assessable on admission and could be assessed by paramedics were used as candidate covariates in multivariate regression.

In the light of collinearity, among the candidate variables that showed high correlation coefficient (Spearman’s r > 0.7) with others, one factor was selected as covariate in the multivariate model and the others were removed. A parsimonious model (model 2) was also made by using predictive factors that showed a significant difference by multivariate logistic regression analysis of model 1. The probability of falls was estimated using the logistic regression model.

The fall rate, sensitivity, specificity, positive predictive value, and negative predictive value of the scores were calculated for the validation set. The cutoff values for scores were set at sensitivity of 90%, specificity of 90%, or maximum sum of sensitivity and specificity in the test set. The predictive performance of the models was assessed by the AUC derived from the validation set. The calibrations of model 1 and model 2 were assessed by shrinkage coefficient [24] and Akaike’s information criteria (AIC) [25].

Analyses were performed using SAS version 9.4 (SAS Institute, Cary, NC, USA) and R version 3.6.0 (R Foundation, Vienna, Austria) with the library “ctree.”

### Ethical considerations

This study conforms to the ethical guidelines for medical and health research involving human subjects issued by the MHLW and the Ministry of Education, Culture, Sports, Science, and Technology in Japan. This study was approved by the research ethics committee of the Yuai-kai Foundation and Oda Hospital (No. 20150910). We obtained consent from all patients by a comprehensive agreement method in the hospital, and anonymity of patients was protected.

## Results

### Patients’ background and incidence of fall events

**Fig 2:**
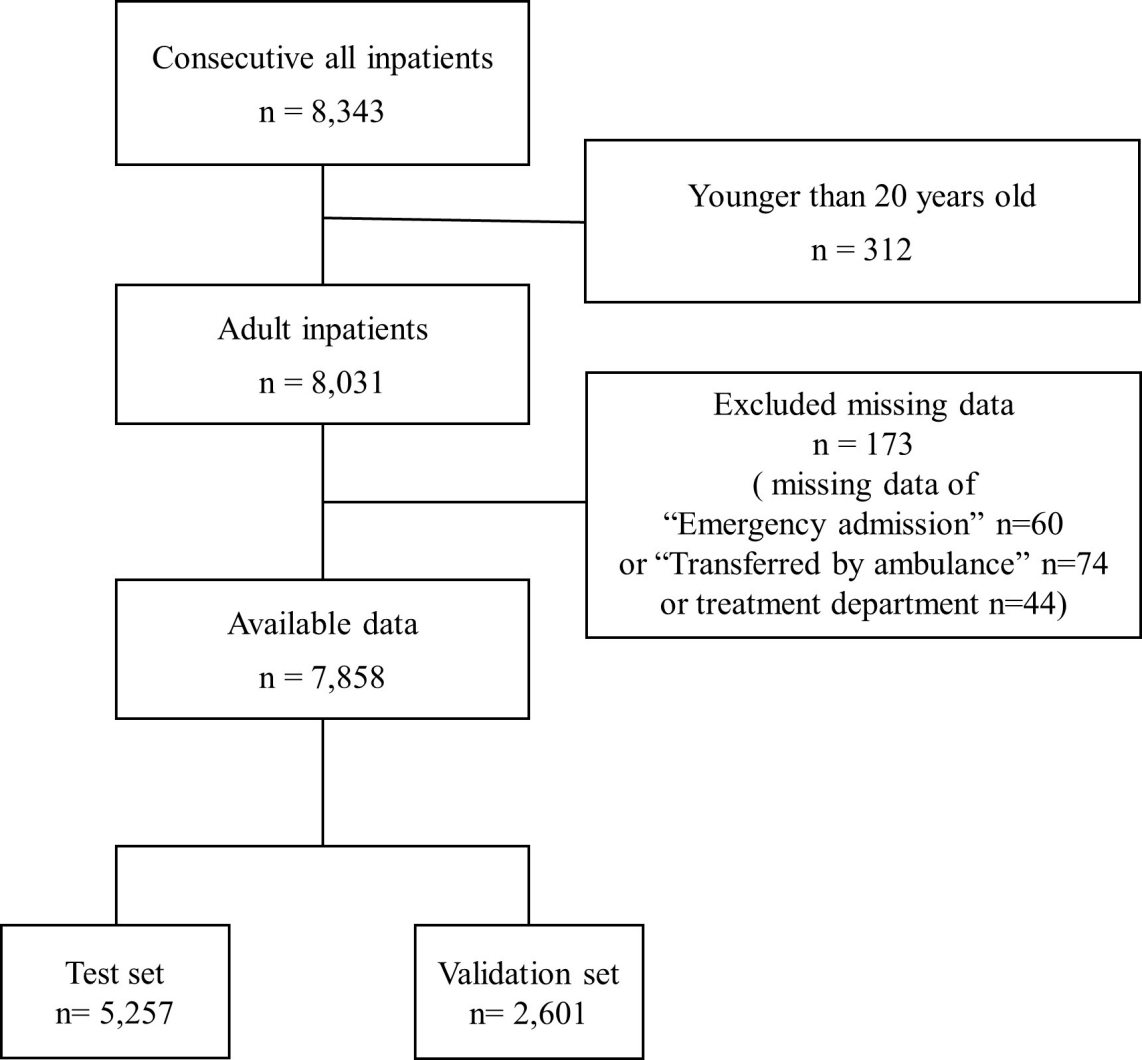
Data flow diagram. A total of 7,858 were eligible, being randomly extracted 1/3 from all cases; the test set, n = 5,257, and the validation set, n = 2,601.

During the study period 8,343 inpatients were admitted, 8,031 of whom were aged 20 years or older. Among them, 7,858 were eligible (Fig 2), being randomly extracted to two groups, the test set (5,257) and the validation set (2,601). In the test set, 243 falls occurred (4.6%), the median age (interquartile range) was 77 years (62–85), 34% were men, the median length of hospital stay (interquartile range) was 9 (5–16) days, and the incidence rate of falls was 3.5 per 1,000 patient-days. In the validation set, 122 falls occurred (4.7%).

### Candidates of predictive factors for fall events

Table 1 shows a comparison of backgrounds of patients with and without falls in both the test set and validation set. Statistical significances are shown between for patients with and without falls for all factors compared except male, transfer by ambulance, presence of referral letter, admission to Department of Internal Medicine, visual impairment, and parkinsonism.

**Table 1:** Patients’ backgrounds with or without fall and their comparison in the test set and validation set.

Variable, Category (Reference)	Test set			Validation set		
	With Fall	Without fall	*P-*value^†^	With Fall	Without fall	*P*-value^†^
	n = 243	n = 5,014		n = 122	n = 2,479	
Age, years	83 (78–88)	76 (62–85)	<0.001	83 (78–87)	76 (62–84)	<0.001
Gender, Male (Female)	140 (57.6)	2,461 (49.1)	0.006	56 (45.9)	1,201 (48.4)	0.324
Emergency admission, Yes (No)	212 (87.2)	3,233 (64.5)	<0.001	104 (85.2)	1,589 (64.1)	<0.001
By ambulance, Yes (No)	39 (16.0)	679 (13.5)	0.155	21 (17.2)	301 (12.1)	0.069
Referral medical letter, Presence (Absence)	92 (37.9)	1,632 (32.5)	0.050	46 (37.7)	779 (31.4)	0.089
Department, Internal Medicine (Others)	29 (11.9)	758 (15.1)	0.100	16 (13.1)	409 (16.5)	0.196
Department, Neurosurgery (Others)	24 (9.9)	183 (3.6)	<0.001	14 (11.5)	88 (3.5)	<0.001
Hypnotic, Using (Not using)	50 (20.6)	540 (10.8)	<0.001	31 (25.4)	265 (10.7)	<0.001
Hypnotic, Missing category	16 (6.6)	455 (9.1)		9 (7.4)	220 (8.9)	
Permanent damage by stroke, Presence (Absence)	26 (10.7)	312 (6.2)	0.007	11 (9.0)	140 (5.6)	<0.001
History of falls, Presence (Absence)	67 (27.6)	486 (9.7)	<0.001	28 (23.0)	243 (9.8)	<0.001
Visual impairment, Presence (Absence)	8 (3.3)	104 (2.1)	0.203	6 (4.9)	55 (2.2)	0.064
Parkinsonism, Presence (Absence)	2 (0.8)	48 (1.0)	0.591	1 (0.8)	17 (0.7)	0.580
Eating, Independent (Requiring assistance)	176 (72.4)	2,611 (52.1)	<0.001	83 (68.0)	1,297 (52.3)	<0.001
Eating, Missing category	14 (5.8)	1,715 (34.2)		9 (7.4)	832 (33.6)	
Toileting, Independent (Requiring assistance)	144 (59.3)	2,383 (47.5)	<0.001	70 (57.4)	1,179 (47.6)	<0.001
Toileting, Missing category	14 (5.8)	1,715 (34.2)		9 (7.4)	832 (33.6)	
Bathing, Independent (Requiring assistance)	112 (46.1)	2,101 (41.9)	<0.001	51 (41.8)	997 (40.2)	<0.001
Bathing, Missing category	14 (5.8)	1,715 (34.2)		9 (7.4)	832 (33.6)	
Taking prescription drug, Independent (Requiring assistance)	112 (46.1)	2,101 (41.9)	<0.001	55 (45.1)	1,036 (41.8)	<0.001
Taking prescription drug, Missing category	14 (5.8)	1,715 (34.2)		9 (7.4)	832 (33.6)	
Transferring, Independent (Requiring assistance)	137 (56.4)	2,211 (44.1)	<0.001	68 (55.7)	1,093 (44.1)	<0.001
Transferring, Missing category	14 (5.8)	1,715 (34.2)		9 (7.4)	832 (33.6)	
Wheelchair, Not using (Using)	26 (10.7)	192 (3.8)	<0.001	12 (9.8)	85 (3.4)	<0.001
Wheelchair, Missing category	48 (19.8)	2,301 (45.9)		22 (18.0)	1,109 (44.7)	
Bedriddenness rank, J (Normal)	27 (11.1)	495 (9.9)	<0.001	10 (8.2)	261 (10.5)	<0.001
Bedriddenness rank, A	83 (34.2)	760 (15.2)		36 (29.5)	374 (15.1)	
Bedriddenness rank, B	63 (25.9)	523 (10.4)		39 (32.0)	237 (9.6)	
Bedriddenness rank, C	46 (18.9)	506 (10.1)		23 (18.9)	250 (10.1)	
Bedriddenness rank, Not assessable	3 (1.2)	223 (4.4)		2 (1.6)	111 (4.5)	
Cognitive function, 1 (Normal)	53 (21.8)	508 (10.1)	<0.001	22 (18.0)	271 (10.9)	<0.001
Cognitive function, 2	53 (21.8)	405 (8.1)		33 (27.0)	213 (8.6)	
Cognitive function, 3	43 (17.7)	421 (8.4)		17 (13.9)	177 (7.1)	
Cognitive function, 4	25 (10.3)	191 (3.8)		11 (9.0)	97 (3.9)	
Cognitive function, M	6 (2.5)	29 (0.6)		1 (0.8)	15 (0.6)	
Cognitive function, Missing category	4 (1.6)	235 (4.7)		2 (1.6)	116 (4.7)	
Fall preventive movement sensor, Using (Not using)	155 (63.8)	636 (12.7)	<0.001	80 (65.6)	281 (11.3)	<0.001
Fall preventive movement sensor, Missing category	22 (9.1)	2,781 (55.5)		4 (3.3)	1,397 (56.4)	
Low bed, Using (Not using)	29 (11.9)	142 (2.8)	<0.001	14 (11.5)	60 (2.4)	<0.001
Low bed, Missing category	22 (9.1)	2,781 (55.5)		4 (3.3)	1,397 (56.4)	
Impact-absorbing mat, Using (Not using)	20 (8.2)	43 (0.9)	<0.001	13 (10.7)	25 (1.0)	<0.001
Impact-absorbing mat, Missing category	22 (9.1)	2,781 (55.5)		4 (3.3)	1,397 (56.4)	
Surgical operation, Done (Undone)	176 (72.4)	1,776 (35.5)	<0.001	89 (73.0)	863 (34.8)	<0.001
Rehabilitation, Done (Undone)	46 (18.9)	1,452 (29.0)	0.001	23 (18.9)	731 (29.5)	0.001
Duration of hospitalization (No reference)	23 (14–40)	9 (4–16)	<0.001	24 (14–41)	9 (5–16)	<0.001
Outcome, Deceased (Alive)	46 (18.9)	141 (2.8)	<0.001	15 (12.3)	81 (3.3)	<0.001

Continuous and categorical variables are presented as median (interquartile range) and frequency (percent). Bedriddenness rank: J, independence/autonomy; A, house-bound; B, chair-bound; C, bed-bound.

^†^*P*-values were calculated by Wilcoxson’s rank-sum test for continuous variables and chi-squared test for categorical variables.

### Predictive models for predicting falls

Table 2 shows the results of multivariate logistic regression analysis of two models. Thirteen predictive factors in model 1 were selected considering collinearity and usability from all 20 factors assessable on admission (S2 Appendix). We removed parkinsonism from eligible factors because of difficulty in precise assessment of the degree of functional impairment from only medical charts in this retrospective study. We performed multivariate logistic regression analysis using 13 factors as follows: age, sex (male), emergency admission, use of ambulance, referral letter, admission to Department of Neurosurgery, admission to Department of Internal Medicine, use of hypnotic medication, permanent damage by stroke, history of falls, visual impairment, independence of eating, and bedriddenness rank. Factors with statistical significance by multivariate analysis in model 1 were sex (male), emergency admission, admission to Department of Neurosurgery, use of hypnotic medication, history of falls, independence of eating, and bedriddenness rank. Age and these seven factors, with a significant difference shown by the multivariate logistic regression analysis of model 1, were used for the multivariate logistic regression analysis of model 2. Consequently, all the factors other than age showed a significant difference.

**Table 2:** Multivariate logistic regression analysis for falls according to the two models.

Variable, Category (Reference)	Multivariate logistic regression using 13 factors (model 1)			Multivariate logistic regression using 8 factors (model 2)		
	OR	95% CI	*P*-value^†^	OR	95% CI	*P*-value^†^
Age category, ≥75 (<75)	1.0	1.0–1.0	0.169	1.0	1.0–1.0	0.146
Gender, Male (Female)	1.8	1.4–2.4	<0.001	1.8	1.3–2.3	<0.001
Emergency admission, Yes (No)	1.6	1.1–2.5	0.018	1.6	1.0–2.3	0.033
By ambulance, Yes (No)	0.8	0.5–1.1	0.214			
Referral medical letter, Presence (Absence)	1.2	0.9–1.5	0.321			
Department, Internal Medicine (Others)	1.2	0.8–1.8	0.421			
Department, Neurosurgery (Others)	2.1	1.3–3.4	0.003	1.9	1.2–3.1	0.008
Hypnotic, Using (Not using)	1.5	1.0–2.0	0.033	1.4	1.0–2.0	0.038
Hypnotic medicine, Missing category (Not using)	1.2	0.7–2.2	0.566	1.3	0.7–2.3	0.484
Permanent damage by stroke, Presence (Absence)	0.8	0.5–1.2	0.275			
History of falls, Presence (Absence)	1.5	1.1–2.1	0.008	1.5	1.1–2.1	0.008
Visual impairment, Presence (Absence)	0.9	0.4–2.0	0.848			
Eating, Independent (Requiring assistance)	1.3	0.9–1.9	0.204	1.3	0.9–1.8	0.214
Eating, Missing category (Requiring assistance)	0.4	0.2–0.7	0.002	0.4	0.2–0.7	0.002
Bedriddenness rank, J (Normal)	4.0	2.2–7.3	<0.001	4.0	2.2–7.2	<0.001
Bedriddenness rank, A (Normal)	6.4	3.8–11	<0.001	6.3	3.7–11	<0.001
Bedriddenness rank, B (Normal)	7.2	4.1–13	<0.001	6.8	3.9–12	<0.001
Bedriddenness rank, C (Normal)	6.1	3.3–11	<0.001	5.6	3.1–10	<0.001
Bedriddenness rank, Not assessable (Normal)	0.9	0.2–3.7	0.907	0.9	0.2–3.4	0.830

OR, odds ratio; CI, confidence interval. Bedriddenness rank: J, independence/autonomy; A, house-bound; B, chair-bound; C, bed-bound. Twelve factors using model 1 were all assessable at admission and had low collinearity with each other. Model 2 was designed as a parsimonious model using eight factors that had significance by multivariate logistic regression of model 1.

^†^*P*-value for Wald test.

### Performance of predictive models

**Fig 3:**
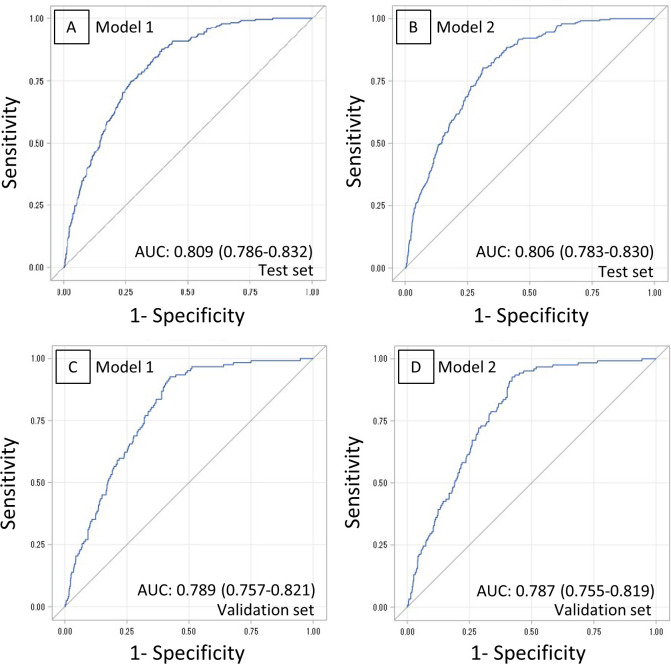
Receiver-operating characteristics (ROCs) and areas under the curves (AUCs). ROC derived from test set using model 1 (A), derived from test set using model 2 (B), derived from validation set using model 1 (C), and derived from validation set using model 2 (D). The AUCs using models 1 and 2, the test set, and the validation set are all above 0.7, and the discrimination ability of the models is good.

**Fig 4:**
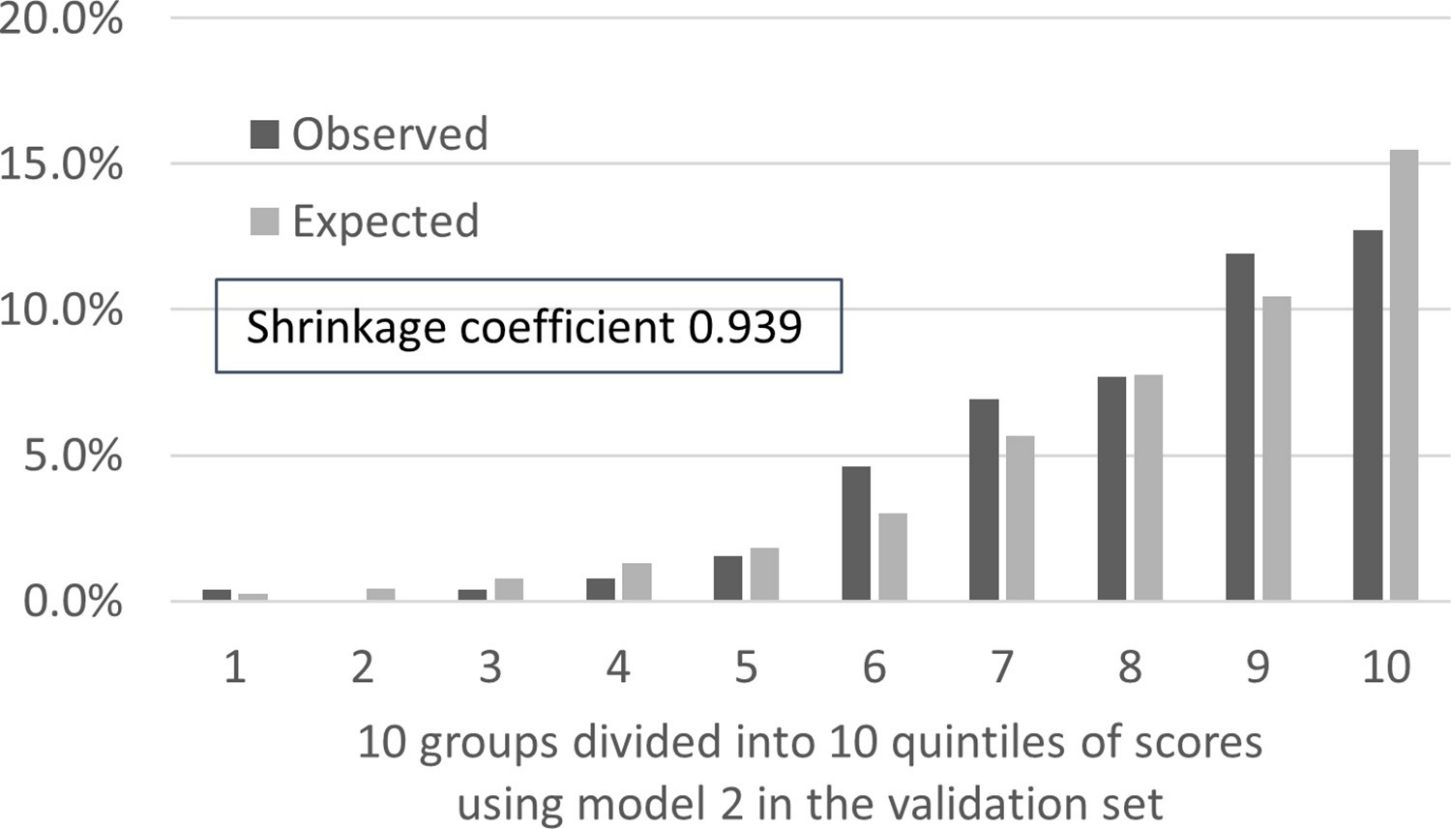
The predicted and observed rates of falls in 10 groups divided into 10 quintiles of scores using model 2. The gap between the predicted and observed values was not large for either group, and the calibration of model 2 was good.

The detailed formula is shown in S3 Appendix. AUC derived from the test set as the predictive performance of model 1 was 0.808 (95% CI: 0.785–0.831; Fig 3A), and AUC as predictive performance of model 2 was 0.806 (95% CI: 0.783–0.829; Fig 3B). Additionally, the AIC of model 1 was 1,706 and that of model 2 was 1,701. The incidence of falls actually observed was consistent with the predicted incidence calculated using model 2, with the shrinkage coefficient of 0.939 (Fig 4).

AUC for the validation set, as the predictive performance of model 1, was 0.789 (95% CI: 0.757–0.821, Fig 3C), and AUC as predictive performance of model 2 was 0.787 (95% CI: 0.755–0.819; Fig 3D). The sensitivity and specificity of model 1 and 2 derived from the test set and validation set with 3 cutoff score was shown in S1 Table.

## Discussion

Incidence of falls estimated per 1,000 days have been reported to have increased by 46% during the last half century [26]. Furthermore, as falls have been reported to have occurred with the highest frequency on the day of admission [27], it is essential to assess patients as soon as possible after their admission and predict and prevent this potentially devastating event. In this study, we developed new and easier-to-use predictive models using 13 or 8 much more accessible items acquired routinely on admission of patients to a Japanese hospital, including the Japanese public scale of bedriddenness (MHLW bedriddenness rank), which we confirmed to have statistical significance. The higher usability of these models derives from the fact that previously developed models required more complicated examinations and techniques seldom used in routine clinical settings in Japan as well as time-consuming and cumbersome assessments, causing a serious impediment to their use. For example, the HFRM has not commonly been used in Japan, although it is one of the more widely used predictive models of falls worldwide [28]. This could partly be related to the fact that this model requires relatively bothersome and too many formal examinations such as the Mini-Mental State Examination [29], Koenig II Depression Rating Scale [30], get-up-and-go test [31], and Bender Elimination [19]. Conversely, our predictive model is extraordinarily convenient and easy to use even in extremely busy clinical situations because it requires only readily available variables such as age, sex, or circumstances of admission, all of which can be easily obtained from hospital charts, in addition to information routinely checked as risk factors on admission such as the use of hypnotic medications or history of previous falls. Of course the Japanese official scale of bedriddenness, the MHLW bedriddenness rank, is also easy to assess [22].

As mentioned above, we used the official MHLW bedriddenness rank as one of the items of our predictive model [22]. Although there are no previous reports of the relationship between bedriddenness rank and incidence of falls, it promises to be a comprehensive evaluation tool integrating many possible risk factors of falls [11, 12]. Bedriddenness was reported to have a moderate relationship with conditions of dependence concerning eating, toileting, and bathing in this and a previous study [32], which were not used as covariates in multivariate analyses in the present study. Additionally, underlying disorders, which were also reported to have some relationship with falls, such as vascular diseases [11], arthritis [11], urinary incontinence [12], and poor hearing [12], were not assessed in this study. However, the coefficient correlation of bedriddenness rank was found to be larger than that of other risk factors in multivariate analysis, which made it a fairly reliable item for predicting falls.

We developed two types of model in this study, model 1 and model 2. Although both models showed almost similar values of AIC and AUC, model 2 requires fewer items (8) than model 1 (13), which made model 2 more usable. With such higher usability, AUC of model 2 derived from the validation set showed a value, 0.79, similar to that of the HFRM derived from an external validation, 0.73, revealing similarly high predictability for falls among inpatients in this study. Furthermore, the sensitivity and specificity of model 2 was 73% and 70% with the cutoff score of −2.78 calculated using the validation set, higher than 70% and 61% of the HFRM calculated using an external validation [20]. Although the discrimination ability of model 2 calculated using the validation set was found to be higher than the actual value, the model is expected to have adequate accuracy.

As another point of note, our study suggested male sex as one of the risk factors of falls, consistent with other reports on inpatients [19, 33, 34] but completely different from some previous reports on community-dwelling people [7, 11]. This difference might arise from the fact that inpatients tend to be constitute to some degree a homogeneous group with impaired physical activities [35] compared with community-dwelling people who live a relatively healthy life [36]. The finding of our study that male sex is one of the risk factors for falls suggests that the risk and predictive factors for falls among inpatients have to be considered separately from those of community-dwelling individuals, although the reasons for this difference remain to be clarified.

During this retrospective observational study, appropriate precautions were taken to prevent patients from falling, such as the use of low beds and sensors detecting patients’ leaving beds without notice, mandatory notification to nurses by inpatients when going to the bathroom, prescribing appropriate rehabilitation during the hospital stay, or recommendation of wearing shoes with a grip sole. These precautions could have had some influence on the results. Moreover, other possible risk factors and etiology of falls, including syncope and hypotension [13], were not assessed in this study because they were not registered in the medical charts. Although parkinsonism, including Parkinson disease, is considered to be one of the essential causal factors of falls, we were forced to remove it from the eligible factors because it was impossible to assess the degree of functional impairment from only medical charts in this retrospective study. Additionally, although the MHLW bedriddenness rank assessed by a nurse in charge of a patient on admission was reassessed by another nurse in charge of preparing discharge of the patient from the hospital, concordance between both evaluations was not verified. Furthermore, because MHLW bedriddenness rank is an ADL indicator used only in Japan, its recognition worldwide is not particularly high, so its correlations with other ADL indicators such as Berthel Index, Katz Index, and DASC-21 should be verified. Additionally, inter-rater reliability, for which HFRM [37] and TMT [38] had shown excellent results, was not assessed in our models. Finally, because the validity of our model was only assessed using the same population as was used in the multivariate analysis, the results could be different when using other groups, requiring assessment of the robustness of the model.

## Conclusion

In conclusion, we developed new and accurate predictive models for falls in adult inpatients in the acute care setting using the public scale of ADL in Japan, the MHLW bedriddenness rank, which has much simpler and fewer predictors than previously reported models.

## Supporting information

S1 Table Validation of model 1 and model 2. (DOCX)

S1 Appendix Characteristics of Yuai-kai Foundation and Oda Hospital. (DOCX)

S2 Appendix Correlation coefficient. (DOCX)

S3 Appendix Formula of models and predictive occurrence. (DOCX)

## Abbreviations

ADLs: activities of daily living
HFRM: Hendrich II Fall Risk Model
MHLW: Ministry of Health, Labour and Welfare
AUC: area under the receiver-operating characteristic curve
AIC: Akaike's information criterion
