# Correction: New predictive models for falls among inpatients using public ADL scale in Japan: A retrospective observational study of 7,858 patients in acute care setting

**DOI:** 10.1371/journal.pone.0307714

**Published:** 2024-07-18

**Authors:** Masaki Tago, Naoko E. Katsuki, Yoshimasa Oda, Eiji Nakatani, Takashi Sugioka, Shu-ichi Yamashita

In S3 Appendix, there is an error in formula of model 2. Please view the correct S3 Appendix below.

## Supporting information

S3 Appendix (DOCX)
