# Supplementary material for: New predictive models for falls among inpatients using public ADL scale in Japan: A retrospective observational study of 7,858 patients in acute care setting
Source: PLoS One. 2020 Jul 16;15(7):e0236130. doi: 10.1371/journal.pone.0236130 (PMC7365416; doi:10.1371/journal.pone.0236130)
Supplement: S1 Table — (DOCX) [file pone.0236130.s001.docx]

**S1 Table** Validation of model 1 and model 2

| Statistics for 3 cutoff points | Model 1 | | Model 2 | |
| --- | --- | --- | --- | --- |
|  | n=5,257 | | n=2,601 | |
|  | Test set | Validation set | Test set | Validation set |
| Cutoff value for scores | −3.66 | −3.66 | −3.80 | −3.80 |
| Probability^†^ | 2.5 |  | 2.2 |  |
| Sensitivity | 90 | 93 | 90 | 93 |
| Specificity | 57 | 57 | 56 | 56 |
| Positive predictive value | 9.3 | 9.7 | 9 | 4.4 |
| Negative predictive value | 99 | 99 | 99 | 99 |
| Cutoff value for scores | −2.74 | −2.74 | −2.78 | −2.78 |
| Probability^†^ | 6.1 |  | 5.8 |  |
| Sensitivity | 78 | 71 | 81 | 73 |
| Specificity | 71 | 71 | 69 | 70 |
| Positive predictive value | 11 | 11 | 11 | 10 |
| Negative predictive value | 99 | 98 | 99 | 98 |
| Cutoff value for scores | −2.03 | −2.03 | −2.01 | −2.01 |
| Probability^†^ | 11.6 |  | 11.8 |  |
| Sensitivity | 37 | 33 | 38 | 30 |
| Specificity | 90 | 90 | 90 | 91 |
| Positive predictive value | 15 | 14 | 16 | 13 |
| Negative predictive value | 97 | 97 | 97 | 96 |

^†^The value was calculated as the probability of a fall for patients with defined score.

Three cutoff points were determined by the minimum score over 90% sensitivity, the optimal point by Youden index, and the maximum score over 90% specificity.

The sensitivity and specificity of model 1 derived from the test set were 90% and 57% with the cutoff score of −3.66, 78% and 71% with −2.74, and 37% and 90% with −2.03, respectively. In similar fashion, the sensitivity and specificity of model 2 derived from the test set were 90% and 56% with the cutoff score of −3.80, 81% and 69% with −2.78, and 38% and 90% with −2.01, respectively.

The sensitivity, specificity, positive predictive value, and negative predictive value of model 1 were 93%, 57%, 9.7%, and 99% with the cutoff score of −3.66, 71%, 71%, 11%, and 98% with −2.74, and 33%, 90%, 14%, and 97% with −2.03, respectively. In similar fashion, the sensitivity, specificity, positive predictive value, and negative predictive value of model 2 were 93%, 56%, 4.4%, and 99% with the cutoff score of −3.80, 73%, 70%, 10%, and 98% with −2.78, and 30%, 91%, 13%, and 96% with −2.01, respectively.
