# Supplementary material for: New predictive models for falls among inpatients using public ADL scale in Japan: A retrospective observational study of 7,858 patients in acute care setting
Source: PLoS One. 2020 Jul 16;15(7):e0236130. doi: 10.1371/journal.pone.0236130 (PMC7365416; doi:10.1371/journal.pone.0236130)
Supplement: S1 Appendix — (DOCX) [file pone.0236130.s002.docx]

**Characteristics of Yuai-kai Foundation and Oda Hospital**

The hospital comprises 10 departments with 111 beds, including the Departments of Internal Medicine, General Surgery, and Cardiovascular Surgery but not of Orthopedic Surgery. The hospital is located in a suburban city, Kashima, in Saga prefecture in southern Japan, covering an approximate population of 90,000, annually treating over 3,100 inpatients with a mean length of stay of 12.1 days.
