# Supplementary material for: New predictive models for falls among inpatients using public ADL scale in Japan: A retrospective observational study of 7,858 patients in acute care setting
Source: PLoS One. 2020 Jul 16;15(7):e0236130. doi: 10.1371/journal.pone.0236130 (PMC7365416; doi:10.1371/journal.pone.0236130)
Supplement: S2 Appendix — (DOCX) [file pone.0236130.s003.docx]

**Correlation coefficient**

Ability to eat had a strong correlation with ADLs, i.e., the ability to toilet (correlation efficient, r = 0.95), bathe (r = 0.92), take drugs (r = 0.92), transfer (r = 0.93), and use a wheelchair (r = 0.73). Bedriddenness rank had strong correlation with cognitive function score (r = 0.73) and a moderate correlation with ADLs (0.41 ≤ r ≤ 0.63).
