# Supplementary material for: New predictive models for falls among inpatients using public ADL scale in Japan: A retrospective observational study of 7,858 patients in acute care setting
Source: PLoS One. 2020 Jul 16;15(7):e0236130. doi: 10.1371/journal.pone.0236130 (PMC7365416; doi:10.1371/journal.pone.0236130)
Supplement: S3 Appendix — (DOCX) [file pone.0236130.s004.docx]

**Formula of model 1**

−5.9296 + 0.0092 × (Age) + (Male = 0.5918) + (Emergency admission = 0.4965) + (Transferred by ambulance = −0.2391) + (Presence of referral letter = 0.1420) + (Admitted department; Internal Medicine = 0.1714, Neurosurgery = 0.7320) + (Hypnotics; Using = 0.3727, Missing data = 0.1762) + (Permanent residual damage from previous strokes = −0.2486) + (History of fall = 0.4363) + (Visual impairment = −0.0742) + (Ability of eating; Independent = 0.2429, Missing data = −1.0483) + (Bedriddenness rank; J = 1.3878, A = 1.8625, B = 1.9672, C = 1.8003, Not assessable = −0.0825).

**Formula of model 2**

−5.8563 + 0.0096 × (Age) + (Male = 0.5684) + (Emergency admission = 0.4418) + (Admitted department; Neurosurgery = 0.6520) + (Hypnotics; Using = 0.2139, Missing data = 0.3612) + (History of fall = 0.4362) + (Ability of eating; Independent = 0.2352, Missing data = −1.0436) + (Bedriddenness rank; J = 1.3758, A = 1.8317, B = 1.9186, C = 1.7205, Not assessable = −0.1505).

**Predictive occurrence of fall during the administration (%)**

100 × exp(Score)/{1 + exp(Score)}.
